# Practical Algal Control in Lower Yangtze Reservoirs Using Composite Microfiltration Physical Enclosure

**DOI:** 10.3390/membranes15100311

**Published:** 2025-10-13

**Authors:** Bin Xu, Fangzhou Liu, Qi Zhang, Congcong Ni, Jianan Gao, Xin Huang

**Affiliations:** 1 School of Environmental and Chemical Engineering, Shanghai University, Shanghai 200444, China; xubin-shu@shu.edu.cn (B.X.); zhangqi0508@shu.edu.cn (Q.Z.); 2 Shandong Institute for Product Quality Inspection, Jinan 250102, China; liufangzhou2024@126.com; 3 Department of Civil Engineering, The University of Hong Kong, Hong Kong 999077, China

**Keywords:** algal control, composite enclosure, reservoir

## Abstract

Source water reservoirs in the lower reaches of the Yangtze River are increasingly threatened by algal contamination, driven by fluctuations in upstream water quality. To ensure stable reservoir operation and protect downstream drinking water sources, physical enclosures are widely used. However, most algal pollution in reservoirs consists of microalgae (diameters < 100 μm), and conventional algae barriers are effective primarily against visible algal blooms but perform poorly against microscopic algal clusters. To address this limitation, we developed a composite microfiltration physical enclosure system by integrating a microfiltration membrane, supported by a mechanical layer, onto physical enclosures. The algal removal performance of this system was evaluated from lab-scale tests to field-scale applications. Results demonstrated that the composite membrane exhibited excellent interception efficiency against algal aggregates, with algae density in the filtered water reduced by over 80%. The composite enclosure effectively filters multiple algae species, significantly reducing the risk of algae entering downstream water treatment plants, thereby alleviating the burden of traditional processes and reducing operating costs.

## 1. Introduction

Algal blooms (ABs) represent a growing environmental challenge in freshwater reservoirs worldwide, especially in the intensive human–water interacting regions such as the lower Yangtze River basin [1,2,3,4]. Driven by seasonal variations in hydrological conditions, temperature, and nutrient loading from upstream, many drinking water source reservoirs in this area suffer from recurrent algal contamination [5,6]. These blooms degrade water quality through oxygen depletion, pH fluctuation, and the release of odor compounds and cyanotoxins, posing serious threats to aquatic ecosystems and public health [7,8]. The persistent occurrence of microalgae, particularly species with cell diameters below 100 μm, challenges the safety and stability of drinking water supply systems, underscoring the urgency for effective and sustainable algal control strategies [9].

Given the adverse impacts of ABs on both ecological systems and public health, researchers have pursued various strategies to control algal pollution in source waters [10]. These approaches can be broadly classified into two categories: source control and downstream treatment. Source control involves implementing management measures such as restricting pollutant discharge and reducing nutrient levels to inhibit algal growth [11]. However, these methods are often difficult to enforce, require long timeframes to yield results, and may be insufficient under dynamic hydrological conditions [12]. Alternatively, downstream treatment technologies implemented within reservoirs or water treatment plants include biological, chemical, and physical methods. Biological approaches leverage algivorous organisms but face challenges in scalability and efficiency [10,13,14]. Chemical methods, employing algaecides such as copper sulfate or oxidants, risk secondary pollution and the release of intracellular toxins [15,16,17]. Physical methods-including mechanical skimming, air-lift systems, and conventional enclosure barriers-are widely employed for their immediacy and non-toxic nature [18,19,20]. However, most existing physical enclosures are designed primarily to block visible algal mats and floating debris, offering limited retention of unicellular and colonial microalgae that prevail in reservoir environments. Their large pore sizes (typically above 500 μm) fail to intercept microscopic algal clusters, allowing significant penetration and downstream escape. Consequently, there is a critical need for advanced barrier technologies that enhance microalgae removal without compromising operational feasibility or cost-effectiveness.

Microfiltration has gained attention as a promising physical separation technology for algal removal in source water reservoirs, offering advantages such as rapid response, no chemical additives, and minimal ecological impact [21,22,23]. As a simple and cost-effective isolation measure, microfiltration-based enclosures are widely used in lakes and reservoirs to contain pollutants including spilled oil, floating debris, and algal scums. These systems are particularly effective at intercepting visible algal blooms and large aggregates. However, their performance in retaining microscopic algal cells (typically <100 μm) remains inadequate due to the relatively low mechanical strength of conventional barrier materials. Moreover, to date, no studies have systematically evaluated the use of microfiltration-enhanced enclosures for practical algal control in the complex hydrodynamic and ecological environment of the lower Yangtze River reservoirs.

In this study, we designed and implemented a composite microfiltration physical enclosure system to enhance algal retention in real-world reservoir applications. We systematically evaluated the algal interception performance of the system using algal density and chlorophyll-a (Chl-a) as key indicators. The influences of operational parameters-including filtration flux, initial Chl-a level, and turbidity-on algal removal efficiency were investigated. To elucidate the underlying filtration mechanisms, the classic Hermia membrane fouling model was applied to analyze the interception process. Furthermore, the reusability and stability of the system were assessed through cyclic performance tests involving hydraulic cleaning procedures. Our findings demonstrate that the proposed composite microfiltration enclosure offers an efficient, sustainable, and economically viable solution for controlling algal contamination in drinking water sources.

## 2. Materials and Methods

### 2.1. Reagents and Materials

All reagents were of analytical grade and used without further purification unless otherwise specified. Acetone (C_3_H_6_O, analytical grade), Magnesium carbonate (MgCO_3_), Iodine (I_2_), Potassium iodide (KI), Anhydrous ethanol (C_2_H_5_OH), Sodium dihydrogen phosphate dihydrate (NaH_2_PO_4_·2H_2_O), Diatomaceous earth (SiO_2_·nH_2_O) were obtained from Sinopharm Chemical Reagent Co., Ltd. (Shanghai, China). Ultrapure water (H_2_O, 18.2 MΩ·cm) was used in all experimental procedures.

The uses of each reagent are as follows: diatomaceous earth is used to prepare simulated turbidity water samples; sodium dihydrogen phosphate dihydrate is used to adjust the pH value of the solution; magnesium carbonate powder and acetone solution are used for Chl-a spectrophotometric determination; iodine and potassium iodide are used to prepare Luger’s reagent to color algae; and anhydrous ethanol is used for instrument cleaning.

### 2.2. Composite Microfiltration Enclosure

**Scheme 1:**
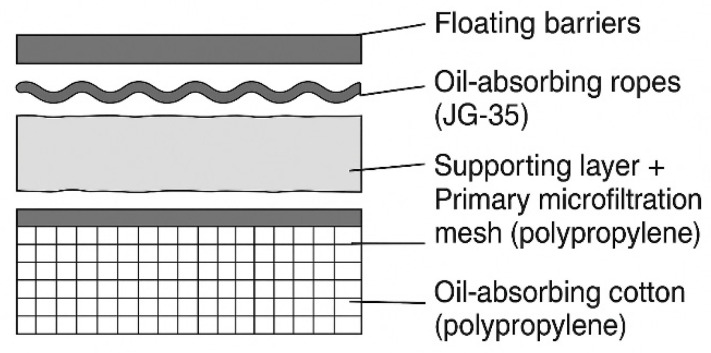
Schematic diagram of composite enclosure.

The composite microfiltration membrane used in this study was provided by Shanghai Chuanxi Technology Co., Ltd. (Shanghai, China) (Figure S1). As schematically illustrated in Scheme 1, the composite microfiltration enclosure system consists of a primary microfiltration mesh (polyethylene), a supporting layer, oil-absorbing ropes (JG-35), oil-absorbing cotton (polypropylene), and floating barriers. The key filtration component is the polyethylene mesh, which is contained within the oil-absorbing layer. Its thickness is 6–8 mm, and its fiber diameter is approximately 20 μm. For lab-scale testing, small sections of the enclosure material were cut into samples measuring 80 mm × 80 mm × 8 mm (Length × Width × Thickness) to fit the experimental filtration setup described in Section 2.3. Notably, the surface pore size cannot be directly measured by SEM due to the oil-absorbing cotton. The pore size of the microfiltration membrane is provided by the manufacturer and is approximately 50–100 μm.

### 2.3. Filtration Experimental Setup

**Figure 1:**
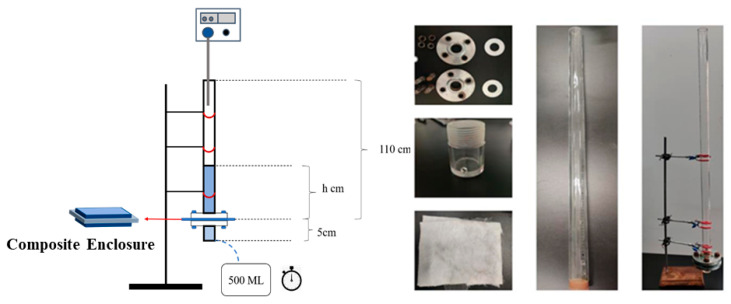
Schematic diagram of filtration experiments.

The enclosure material used in the experiments was sourced from an actual reservoir. As illustrated in Figure 1, the experimental setup consisted of the enclosure clamped between two flanges and sealed with a polytetrafluoroethylene (PTFE) gasket to prevent leakage. The flange type was DN32, with an inner diameter of 42 mm. Two acrylic glass tubes (outer diameter: 42 mm; inner diameter: 32 mm; height: 1.1 m) were connected to both ends of the flange assembly. The bottom of one tube was sealed, and a 6 mm diameter outlet was drilled on its side. A peristaltic pump with a flow rate range of 10–100 mL/min was used to feed the algal suspension into the system. The entire filtration unit was mounted on a metal stand for stability. The filtration flux was determined by measuring the volume of effluent collected in a 500 mL graduated cylinder over one minute. It should be noted that the actual falling water pressure difference in the source reservoir is 0.5 m head, so the water column height was maintained at 0.5 m (0.05 bar) throughout the membrane filtration simulation experiment. The water flux was controlled by adjusting the inlet and outlet flow rates.

For regeneration experiments, the composite microfiltration enclosure was cleaned by backwashing. The setup was identical to that described above, except that the hood was inverted to enable reverse flow. Backwashing was conducted for several minutes at a constant hydrostatic pressure of 0.5 m water head, until the effluent appeared clear. This cleaning procedure was repeated prior to each reuse cycle. All experiments were repeated at least twice.

### 2.4. Evaluation of Algal Interception Efficiency

Microcystis aeruginosa (strain No. FACHB-315, purchased from the Institute of Hydrobiology, Wuhan, China) was cultured in the laboratory. Synthetic algal water was prepared with Chl-a concentrations ranging from 10 to 40 μg/L, reflecting typical conditions in source water reservoirs [24]. To simulate natural turbidity, diatomite was sieved through a 200-mesh screen and added to the algal suspension under continuous stirring to achieve stable turbidity levels between 10 and 60 NTU. All experiments were repeated at least twice.

The filtration flux was controlled between 0.001 and 0.05 m^3^/m^2^·s. Each experiment was terminated when the water level difference across the enclosure exceeded 0.5 m. Effluent samples were collected every 30 min for the determination of Chl-a concentration and turbidity. The interception efficiency was calculated using Equation (1):

Interception Efficiency (%) = (C_0_ − C_e_)/C_0_ × 100% (1)

where C_0_ and C_e_ represent the Chl-a concentrations in the feed and effluent, respectively.

After each experiment, the intercepted algae were thoroughly rinsed from the enclosure, and the washout was collected for Chl-a measurement to quantify the amount of algae retained. The enclosure was cleaned completely to remove the filter cake, and its reusability was evaluated through repeated filtration cycles.

The total filtration resistance (Rₜ, m^−1^) during operation was calculated using Darcy’s law, as given in Equation (2):

J = ΔP/(μ × Rₜ)(2)

where J is the membrane flux (m^3^/m^2^·s), ΔP is the transmembrane pressure (Pa), and μ is the dynamic viscosity of the feed solution (Pa·s).

### 2.5. Hermia Membrane Fouling Model

The Hermia model was applied to analyze the fouling mechanism during algal interception. This model includes four sub-models: cake filtration, intermediate blocking, standard blocking, and complete blocking. The mathematical expressions and linearized forms of each model are summarized in Table 1.

The flux-time data obtained from the experiments were fitted to each linearized model. The coefficient of determination (R^2^) was used to evaluate the suitability of each model and identify the dominant fouling mechanism.

### 2.6. Chlorophyll-a Determination

Chl-a concentration (μg/L) was determined spectrophotometrically. A 500 mL water sample was filtered through a 0.45 μm membrane under a vacuum pressure of less than 50 kPa to prevent cell rupture. After filtration, the sample was refrigerated and dried for 6–8 h before being transferred to a tissue grinder. A small amount of magnesium carbonate (to prevent acidification of Chl-a) and 2–3 mL of 90% acetone were added, and the sample was thoroughly ground to extract Chl-a. The homogenate was centrifuged at 3000–4000 rpm for 10 min, and the supernatant was transferred to a 10 mL colorimetric tube. The extraction (using 2–3 mL of 90% acetone) and centrifugation were repeated 1–2 times. All supernatants were combined and the volume was made up to 10 mL with 90% acetone. The solution was thoroughly mixed and briefly protected from light before measurement. The absorbance was recorded using a spectrophotometer using a 1 cm pathlength cuvette and a 90% acetone blank. Measurements were taken at 750, 663, 645, and 630 nm. The absorbance at 750 nm was used to correct for turbidity, and the Chl-a concentration was calculated based on the corrected values at 663, 645, and 630 nm using the following equation:

(3) $$\mathrm{Chl-}a\;=\;\frac{\left[11.64\times \left(D_{663}-D_{750}\right)-2.16\left(D_{645}-D_{750}\right)+0.1\times \left(D_{630}-D_{750}\right)\right]\times V_{1}}{V\times \sigma }$$

where *V* is the volume of the sample (L); *D* is the absorbance; *V*_1_ is the volume of the extract after adjustment (mL); σ is the pathlength of the cuvette (cm).

### 2.7. Algal Density Determination

Algal density was determined by the microscopic counting method. Water samples collected from the source were fixed and concentrated by sedimentation. A 500 mL water sample was left to settle for 24 h, after which the supernatant was carefully siphoned off, leaving 20–25 mL of concentrated residue. This residue was transferred into a 30 mL volumetric flask. To minimize sample loss, the original container was rinsed several times with small amounts of the supernatant, and the rinsing solution was also added to the volumetric flask to reach a final volume of 30 mL.

Prior to counting, the microscope was calibrated. Each sample was thoroughly mixed, and 0.1 mL was transferred into a plankton counting chamber. A cover glass was gently placed to avoid air bubbles, completing the preparation of the counting slide. The sample was then examined under a microscope at 100× magnification. A total of 100 fields of view were photographed and analyzed using a MATLAB-based image analysis program (Figure S2, Image J 1.54g). Two slides were counted and the average value was taken. If the counts between the two slides differed by more than 15%, a third slide was prepared, and the average of the two closest values was used. The final algal density was calculated according to Equation (4) and expressed as the number of phytoplankton cells per liter of water.

(4) $$N=\frac{A}{A_{C}}\times \frac{V_{w}}{V}\times n$$

where N is the algae density per liter of water (cells/L); *A* is the total area of the counting chamber (mm^2^); *A_C_* is the counted area (mm^2^), calculated as the field area multiplied by the number of fields observed; *V_W_* is the sample volume after sedimentation and concentration (mL); *V* is the effective volume of the counting chamber (mL); *n* is the number of algae density counted.

### 2.8. Field Deployment and Monitoring

**Scheme 2:**
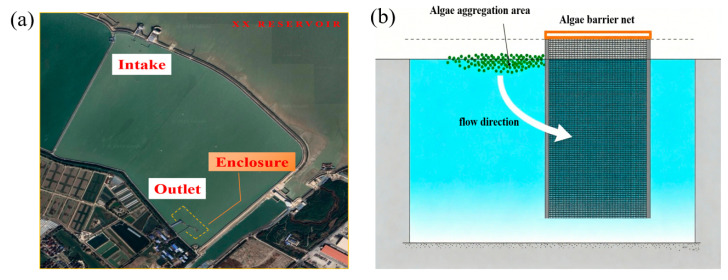
(**a**) Schematic diagram of the deployment location and (**b**) schematic diagram of the structural layout of the composite enclosure in the reservoir.

To evaluate the actual performance of the composite microfiltration enclosure, a field demonstration was conducted at the outlet of a drinking water source reservoir (Reservoir A) in the lower reaches of the Yangtze River (Scheme 2). A containment system approximately 1000 m long was installed. The system consisted of a composite microfiltration mesh (polyethylene, nominal pore size approximately 20 microns, mesh opening 6 cm), a floating barrier, an oil-absorbing rope (JG-35), and oil-absorbing cotton (JGPP-1, polypropylene). The containment was deployed at a depth of approximately 6 m, weighted at the bottom with ceramic weights and secured to the reservoir floor with chains. The bottom of the containment structure was approximately 0.5 m above the sediment to prevent clogging and promote water flow. As part of the reservoir maintenance program, the protective film is replaced every two weeks. Water samples were collected weekly from a depth of 0.5 m upstream (inlet) and downstream (outlet) of the containment structure. The analytical methods for algae density and Chl-a concentration are described in Section 2.6 and Section 2.7.

## 3. Results and Discussion

### 3.1. Current Status of Algal Contamination in Water Sources

**Figure 2:**
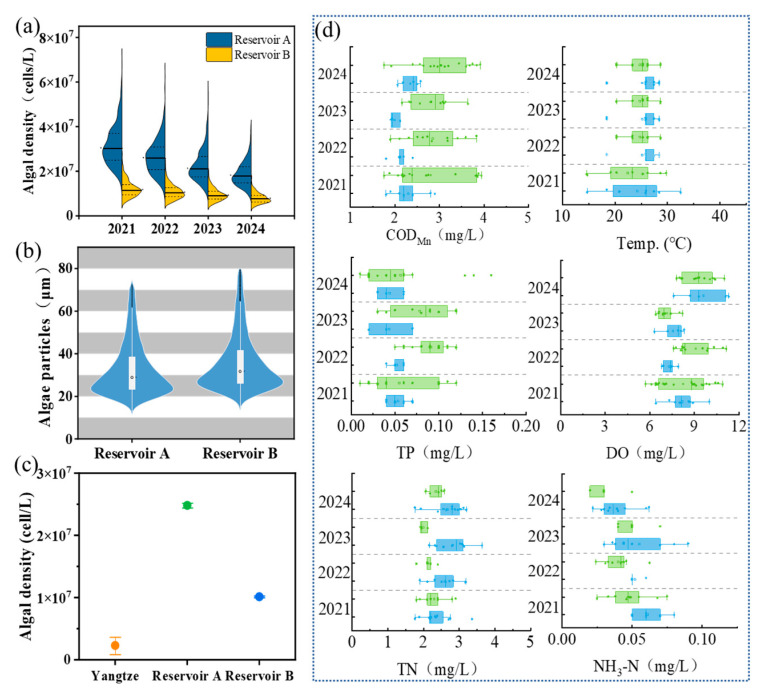
(**a**) Temporal variations in algal density in Reservoirs A and B during 2021–2025. (**b**) Distribution patterns of algal particle size in Reservoirs A and B. (**c**) Comparison of mean algal density between the Yangtze River and Reservoirs A and B. (**d**) Water quality parameters at monitoring sites in Reservoirs A and B.

To evaluate algal contamination in reservoirs located in the lower reaches of the Yangtze River, algal density was continuously monitored in Reservoirs A and B from 2021 to 2024. The results showed that algal concentrations in both reservoirs were generally high, reaching up to 10^7^ cells/L, thereby posing a potential risk to water quality (Figure 2a). The median algal densities in Reservoir A during 2021–2024 were 3.03 × 10^7^ cells/L, 2.95 × 10^7^ cells/L, 2.11 × 10^7^ cells/L, and 1.79 × 10^7^ cells/L, respectively, while those in Reservoir B were 1.15 × 10^7^ cells/L, 1.04 × 10^7^ cells/L, 0.96 × 10^7^ cells/L, and 0.76 × 10^7^ cells/L, respectively. The majority of algal particles were smaller than 100 μm (Figure 2b). Although algal density has been declining annually, reflecting the initial success of various control measures and the continued improvement of water quality in the lower reaches of the Yangtze River, it remains relatively high. As a critical source of urban water supply, the safety of reservoir water quality is directly linked to the stability and security of drinking water supplies.

Notably, algal density in the reservoirs was higher than that in the Yangtze River source water, primarily due to in-reservoir algal proliferation (Figure 2c). Algal growth was largely governed by nutrient levels (total phosphorus, total nitrogen, and ammonia nitrogen) as well as physicochemical factors (pH, permanganate index, dissolved oxygen, and temperature) [25,26,27]. Monitoring data confirmed that all conventional water quality indicators in Reservoirs A and B met the Class II standards of the Surface Water Environmental Quality Standard (GB 3838-2002) (Figure 2d) [28]. Correlation analysis was performed using chlorophyll a concentration as an indicator of algal biomass. Pearson correlation coefficients with environmental factors were calculated using SPSS (IBM SPSS Statistics 28), as summarized in Table 2. At the 0.05 significance level, chlorophyll a in Reservoir A was positively correlated with total phosphorus, total nitrogen, and water temperature but negatively correlated with dissolved oxygen. Similarly, in Reservoir B, chlorophyll a was positively correlated with total phosphorus, ammonia nitrogen, total nitrogen, and water temperature and negatively correlated with dissolved oxygen. The negative correlation between Chl-a and dissolved oxygen (−0.658 and −0.722, respectively) can be attributed to oxygen depletion from algal respiration, whereby higher algal abundance reduces dissolved oxygen [29]. The order of correlation strengths indicated that in Reservoir A, Chl-a was most strongly associated with total nitrogen, followed by water temperature, total phosphorus, and dissolved oxygen, whereas in Reservoir B, the order was total phosphorus > total nitrogen > ammonia nitrogen > water temperature > dissolved oxygen. These results suggest that nutrient concentrations, rather than physicochemical parameters, are the dominant factors driving algal biomass in the reservoirs.

### 3.2. Factors Affecting Composite Microfiltration Enclosure Interception Efficiency

Based on the above analysis of algal contamination and to address the risk of its spread in natural water bodies, this study proposes the application of composite microfiltration enclosure for in-situ algal control (Figure S3). The interception efficiency and its key influencing factors were systematically investigated.

#### 3.2.1. Water Flux

**Figure 3:**
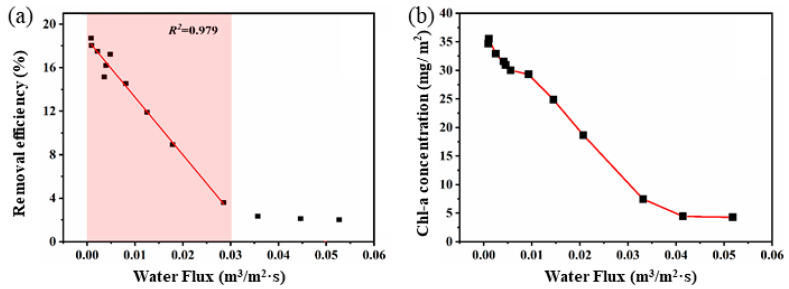
(**a**) Algae removal efficiency of composite microfiltration enclosure under different water fluxes. (**b**) Chlorophyll-a loading on composite microfiltration enclosure at varying water fluxes.

To explore the effect of water flux on interception performance, simulated experiments were conducted with different water fluxes (Figure 3). Flux refers to the volume of water passing through a composite microfiltration enclosure per unit area per unit time. Figure 3a shows that flux significantly affects the interception efficiency of composite microfiltration enclosure for algae: as flux increases, interception efficiency gradually decreases. Within the range of 0–0.03 m^3^/(m^2^·s), flux exhibits a nearly negative linear relationship with interception efficiency (R^2^ = 0.979). Figure 3b shows that as the flow rate increases, the algal load on the composite microfiltration enclosure decreases. When the flow rate exceeds 0.03 m^3^/(m^2^·s), the Chl-a removal efficiency remains low (1–5%). The composite microfiltration enclosure’s removal of algal aggregates is a physical interception mechanism, primarily relying on its pores. When the diameter of the algal aggregates is smaller than the pore size of the composite microfiltration enclosure, the interception effect is poor. Therefore, a lower flow rate favors algal aggregates within the pores, thereby reducing the effective pore size and improving interception capacity. However, excessively high flow rates hinder algal aggregates, resulting in a decrease in interception efficiency.

#### 3.2.2. Turbidity

**Figure 4:**
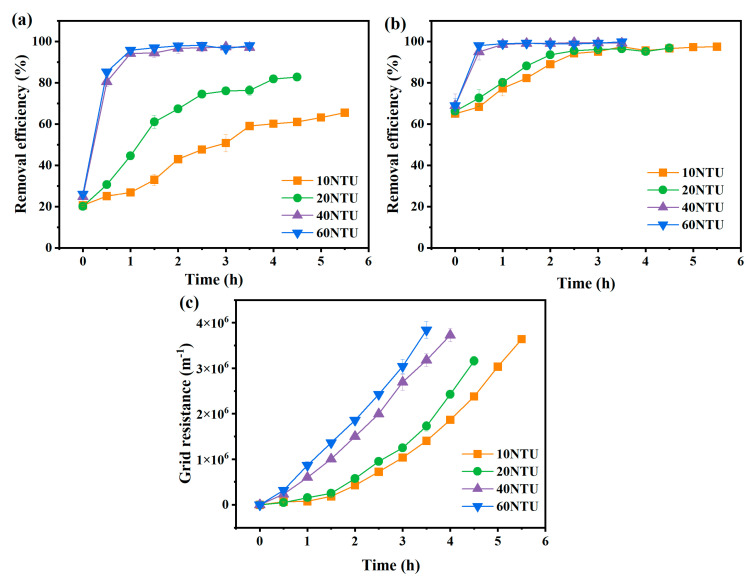
(**a**) Removal efficiency of Chl-a concentration under different turbidity conditions of algae-containing water. (**b**) Interception performance of turbidity by the enclosures. (**c**) Temporal variation of transmembrane resistance of the enclosures under different turbidity conditions.

As the composite microfiltration enclosure primarily relies on physical interception, background turbidity may influence removal efficiency. To simulate actual water conditions, the experiments were conducted within a turbidity range of 10–60 NTU, which is close to the actual conditions in the region’s source reservoirs. As shown in Figure 4a,b, water turbidity significantly enhances the composite microfiltration enclosure’s algal interception effectiveness. Initially, the composite microfiltration enclosure achieved similar Chl-a removal efficiency of approximately 20% for algae-containing water at varying turbidities, while the removal efficiency for turbidity reached 65%. As the experiment progressed, at initial turbidities of 40 NTU and 60 NTU, the Chl-a and turbidity removal efficiencies surged to 80% and 95%, respectively, after 0.5 h, stabilizing after 1 h (approximately 95% for Chl-a and 95% for turbidity). This is because higher turbidity clogs the pores of the composite microfiltration enclosure, forming a continuous filter cake layer that reduces the pore size and significantly increases the interception efficiency [30]. This phenomenon did not occur in the experimental groups with initial turbidities of 10 NTU and 20 NTU, indicating that the filter cake layer was not fully formed under low turbidity conditions, resulting in a slower increase in interception efficiency. It is important to note that while removal efficiency was higher at higher turbidity, the time to reach the experimentally designed 0.5 m head was also shorter. This phenomenon conforms to the “dead-end filtration” kinetic model: the pressure passing through the filter increases linearly, and the resistance of the composite microfiltration enclosure also increases approximately linearly (Figure 4c). Furthermore, higher turbidity increases faster, and the time to reach the set resistance limit is shorter. Overall, within a certain range, higher turbidity increases the composite microfiltration enclosure’s interception efficiency for both algae and turbidity.

#### 3.2.3. Chl-a Concentration

**Figure 5:**
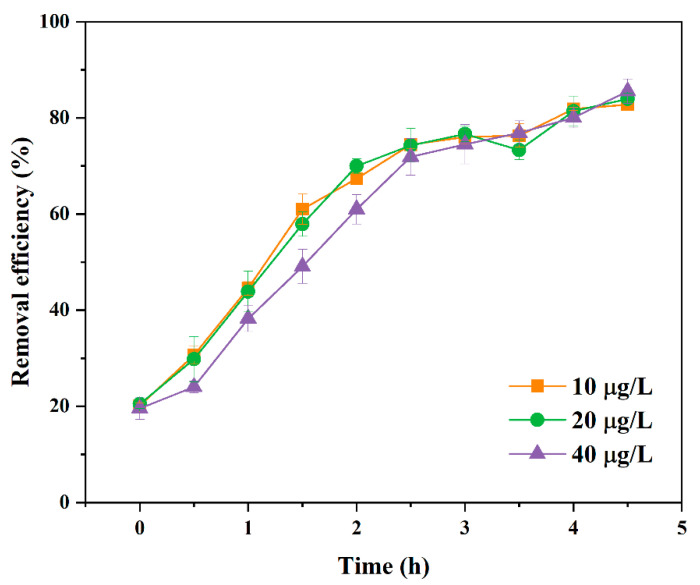
Removal efficiency of Chl-a by the composite microfiltration enclosure at different initial Chl-a concentrations.

This study also examined the removal efficiency of the composite microfiltration enclosure for water bodies with varying degrees of algal contamination. Experiments using algae-containing waters with the same turbidity (20 NTU) and varying Chl-a concentrations revealed that the total amount of algae did not significantly affect the interception efficiency of the composite microfiltration enclosure (Figure 5). The composite microfiltration enclosure maintained similar removal efficiency for water bodies with varying Chl-a concentrations, with no significant differences, indicating that Chl-a concentration is not a critical factor affecting interception efficiency. The composite microfiltration enclosure demonstrated stable and effective algae removal capabilities across varying degrees of algal contamination, making it suitable for remediation of algal contamination at water sources. In summary, water flux and turbidity significantly influence the interception efficiency of composite microfiltration enclosure: the lower the water flux and the higher the turbidity, the better the algae removal effect; Chl-a concentration has a smaller impact. Composite microfiltration enclosure has excellent algae removal potential, but due to practical engineering limitations, it relies primarily on physical isolation to control algae and cannot achieve complete filtration. Therefore, the actual algae removal efficiency is often lower than the theoretical value.

### 3.3. Filtration Mechanism of Composite Microfiltration Enclosure

**Figure 6:**
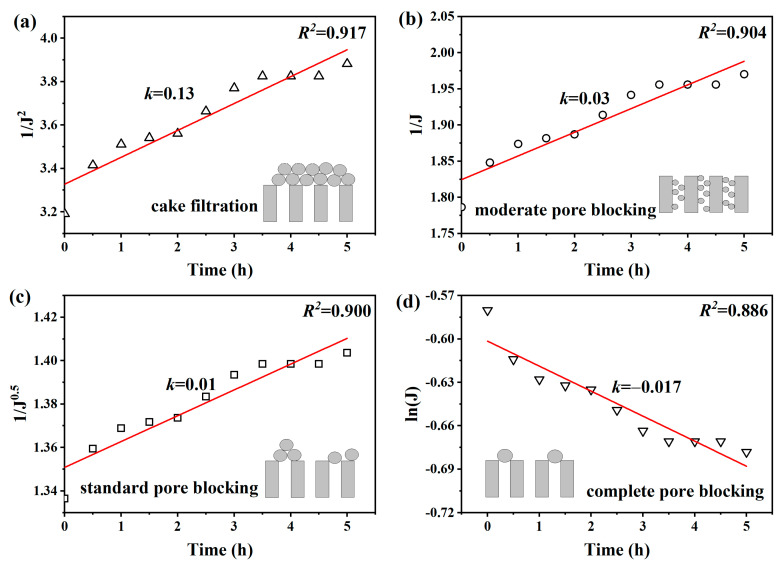
Hermia fouling model fitting for the composite enclosure filtration process: (**a**) cake filtration, (**b**) moderate pore blocking, (**c**) standard pore blocking, and (**d**) complete pore blocking model.

To further elucidate the algal interception mechanism of the composite microfiltration enclosure, the Hermia fouling model was applied to analyze its filtration behavior. Flux decline data over the entire filtration cycle were fitted using the linear forms of four fouling models, and the corresponding fitting constants (k) and coefficients of determination (R^2^) were obtained (Figure 6). The fitting results indicate that the cake filtration model exhibited the highest R^2^ value (0.917), outperforming the intermediate pore blocking (R^2^ = 0.904), standard pore blocking (R^2^ = 0.900), and complete pore blocking (R^2^ = 0.886) models. This quantitative comparison clearly identifies cake filtration as the dominant fouling mechanism. Meanwhile, the intermediate and standard pore blocking models also showed relatively high R^2^ values (>0.9), suggesting that these mechanisms contribute to flux decline in the initial filtration stage, consistent with previous studies [31]. Mechanistically, the cake filtration model exhibited the highest k value (0.13), confirming that rapid deposition of foulants (algal aggregates and suspended particles) on the membrane surface, forming a cake layer, is the primary cause of flux decline [32,33]. In contrast, the k values for intermediate and standard pore blocking were only 0.03 and 0.01, respectively, quantitatively reflecting the minor role of small particles in pore constriction and blockage. As filtration progressed, the filtration resistance increased almost linearly, and the membrane surface became completely covered by particles and algae, eventually forming a mature cake layer. Collectively, these analyses confirm that cake filtration is the predominant mechanism by which the composite microfiltration enclosure intercepts algae.

### 3.4. Composite Microfiltration Enclosure Cleaning and Recycling Capability

**Figure 7:**
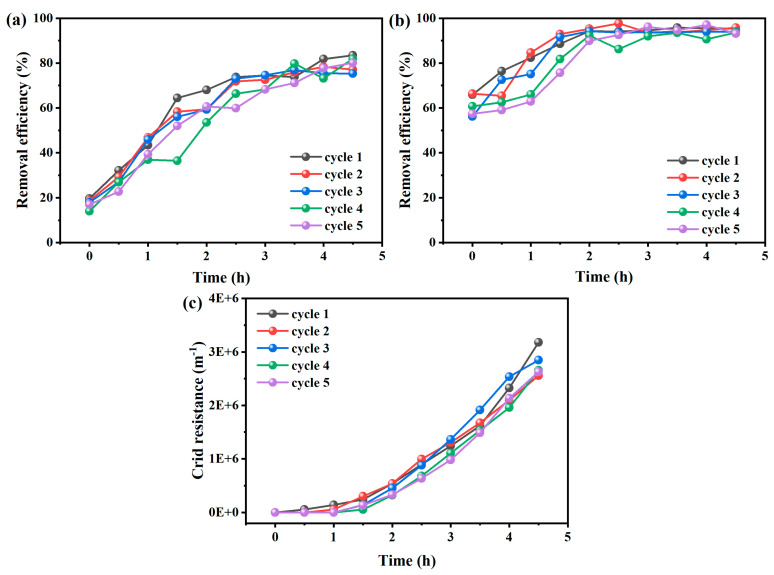
Changes in (**a**) Chl-a removal efficiency, (**b**) turbidity removal efficiency and (**c**) resistance after clean recycling of the composite microfiltration enclosure.

To evaluate this, the enclosures were subjected to backwashing for repeated reuse. As shown in Figure 7, after four cleaning–reuse cycles, the removal efficiencies of Chl-a and turbidity on a single enclosure remained stable. The initial removal efficiencies were 20% and 60%, respectively, which progressively increased during operation and ultimately stabilized above 80% and nearly 100%. Meanwhile, the resistance growth trend remained nearly unchanged across cycles. In addition, the composite enclosure can be regenerated by backwashing and reused in subsequent cycles, reducing material consumption. These results indicate that the composite microfiltration enclosure can be effectively regenerated by backwashing without compromising interception performance or structural integrity, further corroborating that cake filtration is the predominant mechanism. Nevertheless, the retained algal biomass requires safe handling, as cyanotoxin release remains a potential risk under large-scale or long-term use. Future applications should therefore incorporate toxin monitoring and proper biomass management to ensure environmental and public health safety.

### 3.5. Field Performance of the Composite Microfiltration Enclosure in a Reservoir

**Figure 8:**
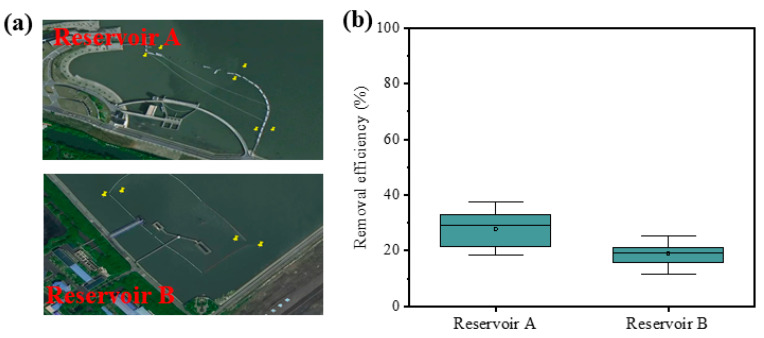
(**a**) Schematic diagram of the enclosure layout and (**b**) actual algae removal efficiency.

To assess the practical algae removal performance of the composite microfiltration cover, it was deployed at a water source in a city in eastern China, and the variation in total algal density before and after installation was monitored (Figure 8a). During operation, conventional water quality indicators (such as turbidity) at Reservoir A were generally consistent with the results of the pilot test (Table S1). However, as shown in Figure 8b, while the device exhibited some retention, its efficiency was significantly lower than observed under laboratory conditions. This discrepancy primarily arose from hydrodynamic limitations. In practice, the actual water head drop at Reservoir A was only 0.5 mH_2_O, while its average daily flow reached as high as 1.6 million tons, corresponding to a flux through the algal net of approximately 0.185 m^3^/m^2^·s. At this pressure, the theoretical maximum flux of the algal net was only 0.075 m^3^/m^2^·s, far below the actual flow rate, indicating that a large portion of the water bypassed the enclosure freely through its bottom. In addition, during sampling, strong winds, high waves, and elevated water levels led to partial submergence of the enclosure, allowing algal-containing water to circumvent the interception zone. Meanwhile, relatively stable downstream hydrodynamic conditions further promoted algal regrowth. These findings highlight that, in practical applications, optimizing the layout of the enclosure and accounting for environmental fluctuations are critical to improving algal removal efficiency.

From an economic perspective, the material cost of the algal net is extremely low. The composite microfiltration cover can continuously retain algae, and only shows a turning point after about two weeks, indicating its practical feasibility (Figure S4). Assuming a cost of approximately USD 10 per square meter, the total cost amounts to about USD 120,000, corresponding to the treatment of ~22.4 million tons of water. This translates to a material cost of only ~USD 0.0025 per ton of water. Although this estimate considers only material expenses while excluding labor and energy inputs, it provides preliminary evidence of the method’s low-cost potential. It should be noted, however, that this result represents a simplified cost analysis; we have accordingly adjusted the discussion of cost-effectiveness in the manuscript to avoid overgeneralization.

## 4. Conclusions

This study developed a composite microfiltration enclosure system for the efficient interception of algal pollution in water source reservoirs along the lower Yangtze River. Experimental results demonstrated that the system exhibited excellent retention performance for algal aggregates, reducing algal density in the filtered water by more than 80%. The system maintained stable algae removal efficiency under varying water quality conditions and could be recycled multiple times through hydraulic backwashing, with cake layer filtration identified as the dominant mechanism. In field applications, the algae removal performance of the enclosure was influenced by environmental fluctuations (e.g., wind, waves, and water level variations) and deployment location (e.g., high-flow zones). Nevertheless, the system effectively removed algae and showed great potential as a high-efficiency, sustainable, and low-cost technology for algal bloom control and drinking water safety assurance in source reservoirs.

## Figures and Tables

**Table 1:** Hermia filtration model.

Filtration Model	Mathematical Expression	Linear Form
Cake filtration	$J_{t}=\sqrt{\frac{1}{\frac{1}{J_{0}^{2}}+kt}}$	$\frac{1}{J^{2}}=\frac{1}{J_{0}^{2}}+kt$
Intermediate blocking	$J_{t}=\beta \frac{1}{1+kt}$	$\frac{1}{J}=\frac{1}{J_{0}}+kt$
Standard blocking	$J_{t}=\frac{J_{0}}{{\left(1+\alpha t\right)}^{2}}$	$\frac{1}{\sqrt{J}}=\frac{1}{\sqrt{J_{0}}}+kt$
Complete blocking	$J_{t}=J_{0}exp(-kt)$	$lnJ=lnJ_{0}-kt$

*Jₜ*: flux at time t (m^3^/(m^2^·s)); *J*_0_: initial flux of clean enclosure (m^3^/(m^2^·s)); *k*, α, β: model constants.

**Table 2:** Correlation coefficients of water quality indicators and Chl-a in different reservoirs.

	TP	NH_3_-N	TN	pH	COD_Mn_	DO	Temp.
**Reservoir A**	0.675 *	0.355	0.776 **	−0.223	0.378	−0.658 *	0.696 *
**Reservoir B**	0.706 *	0.672 *	0.684 *	0.521	0.519	−0.722 *	0.643 *

Noted: * represents *p* < 0.05, ** represents *p* < 0.01.

## Data Availability

Dataset available on request from the authors.

## Supplementary Materials

The following supporting information can be downloaded at: https://www.mdpi.com/article/10.3390/membranes15100311/s1, Figure S1: Actual picture of composite microfiltration physical enclosure; Figure S2: MATLAB program counting schematic; Figure S3: Plan view of the physical enclosure of the composite microfiltration system; Figure S4: Algae density removal efficiency of enclosures during actual operation in the reservoir; Table S1: Median value of conventional water quality indicators of Reservoir A.
