# Supplementary material for: Practical Algal Control in Lower Yangtze Reservoirs Using Composite Microfiltration Physical Enclosure
Source: Membranes (Basel). 2025 Oct 13;15(10):311. doi: 10.3390/membranes15100311 (PMC12566066; doi:10.3390/membranes15100311)
Supplement: Supplementary file 1 [file membranes-15-00311-s001.zip › membranes-3882015-supplementary.pdf]

Supplementary Information for

Practical Algal Control in Lower Yangtze Reservoirs Using Composite Microfiltration Physical Enclosure

Bin Xu [1,†], Fangzhou Liu [2,†], Qi Zhang [1,†], Congcong Ni [1,*], Jianan Gao [3,*] and Xin Huang [1,*]

1 School of Environmental and Chemical Engineering, Shanghai University, Shanghai 200444, China; xubin-shu@shu.edu.cn (B.X.); zhangqi0508@shu.edu.cn (Q.Z.)
2 Shandong Institute for Product Quality Inspection, Jinan 250102, China; liufangzhou2024@126.com (F.L.)
3 Department of Civil Engineering, The University of Hong Kong, Hong Kong 999077, China
* Correspondence: congcongni95@shu.edu.cn (C.N.); jngao@hku.hk (J.G.); huangxin2008@shu.edu.cn (X.H.)
† The authors contribute to this work equally.

Table S1 Median value of conventional water quality indicators of Reservoir A

	**2021**	**2022**	**2023**	**2024**
**DO**	7.83 mg/L	6.71 mg/L	7.14 mg/L	9.02 mg/L
**COD_{MN}**	2.13 mg/L	2.08 mg/L	1.91 mg/L	2.38 mg/L
**NH_3**	0.58 mg/L	0.47 mg/L	0.44 mg/L	0.37 mg/L
**TP**	0.05 mg/L	0.048 mg/L	0.042 mg/L	0.037 mg/L
**Algal density**	3.03×10^7 cell/L	2.59×10^7 cell/L	2.11×10^7 cell/L	1.79×10^7 cell/L
**NTU**	37.2	28.9	41.4	35.8

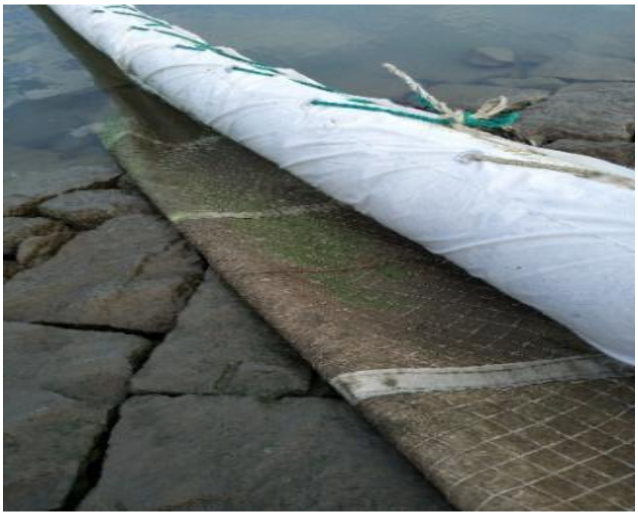

Figure S1 Actual picture of composite microfiltration physical enclosure

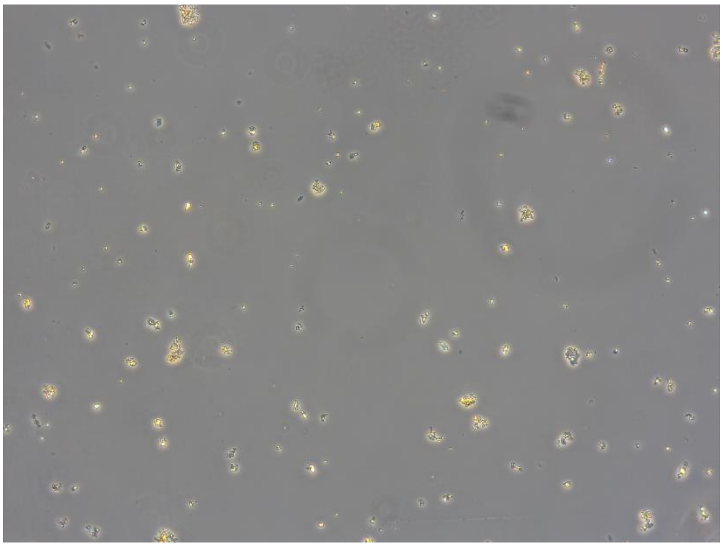

Figure S2 MATLAB program counting schematic

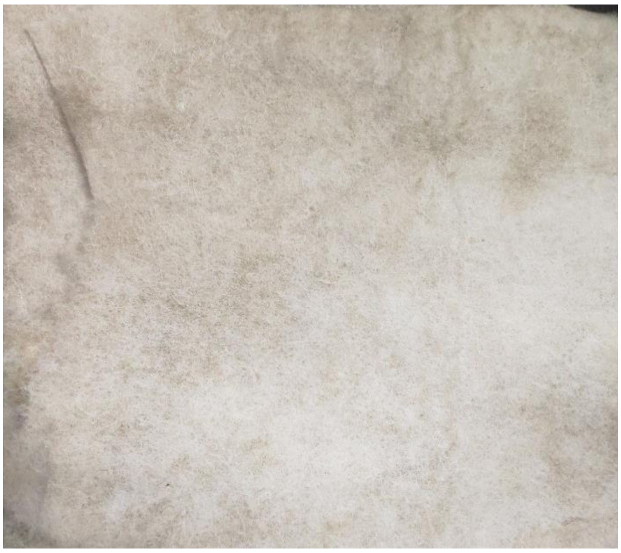

Figure S3 Plan view of the physical enclosure of the composite microfiltration system

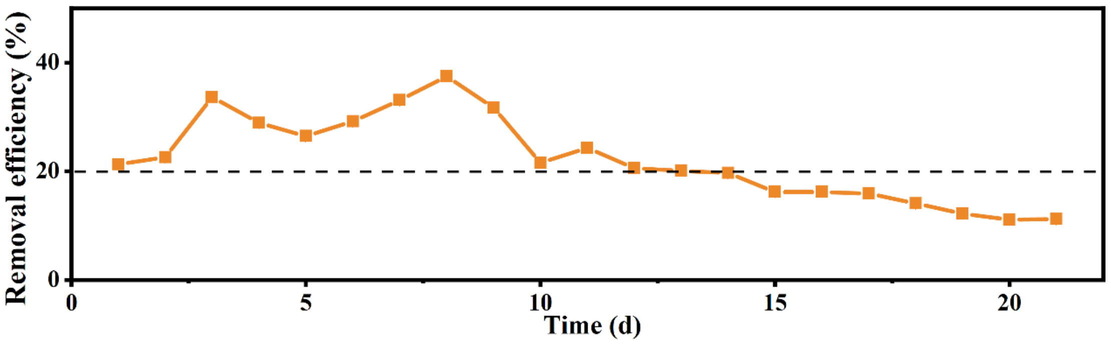

Figure S4 Algae density removal efficiency of enclosures during actual operation in the reservoir
